# A Literature Review and a Proposed Classification of the Relationships between Ovulatory Infertility and Lifestyle Factors Based on the Three Groups of Ovulation Disorders Classified by WHO

**DOI:** 10.3390/jcm12196275

**Published:** 2023-09-28

**Authors:** Magdalena Skowrońska, Michał Pawłowski, Robert Milewski

**Affiliations:** 1 Doctoral Studies, Medical University of Bialystok, 15-089 Bialystok, Poland; magdalena.skowronska@sd.umb.edu.pl; 2 Department of Biostatistics and Medical Informatics, Medical University of Bialystok, 15-089 Bialystok, Poland; michal.pawlowski@umb.edu.pl

**Keywords:** ovulatory infertility, ovulation disorders, lifestyle, diet, physical exercise, insulin resistance, PCOS

## Abstract

Ovulatory infertility is a serious clinical problem whose direct causes are still largely unknown. In addition to pathologies that make it impossible for a couple to establish a pregnancy, there are a number of other factors that have a bearing on fertility, including lifestyle factors, and particularly diet. Although numerous studies have been performed linking such factors to ovulatory infertility, most of them lack the necessary clinical significance, instead focusing on observational data and suggesting or establishing associative relationships. This article consists of a literature review focusing on connections between lifestyle factors such as diet, physical exercise, oxidative stress, sleep, and supplementation, and ovulatory infertility. Special emphasis was given to issues such as obesity and insulin resistance and their mutual relationship with other factors linked to ovulatory infertility. In addition, based on the conclusions of the literature review, the authors have proposed a classification of relationships between ovulation disorders and lifestyle factors in ovulatory infertility within the framework of the WHO classification of ovulation disorders. Furthermore, areas that merit further research have been indicated as well as those that do not. WHO Group II disorders gained prominence in the results of the study as the number of links with lifestyle factors and ovulatory infertility found in the course of the review greatly exceeded those for Groups I and III. The data presented in the article show that the issues of proper diet and physical exercise are those that could benefit from robust clinical studies focused specifically on ovulation infertility, while studies concerning the relationship between oxidative stress, sleep, and supplementation and ovulatory infertility do not seem to be promising directions as far as clinical significance is concerned.

## 1. Introduction

Infertility and fertility disorders are prevalent in a large proportion of the population. Globally, the incidence of infertility is approx. 50–70 million of couples [1]. Infertility is defined as failure to establish a clinical pregnancy after 12 months of regular unprotected sexual intercourse [2]. In the majority of cases, infertility may be attributed to the male, the female, or the combined factor, with numerous causes or risk factors identified as connected with infertility. Female infertility may result from ovarian insufficiency, diminished ovarian reserve (DOR), fallopian tube obstruction, or the abnormal anatomy of the female reproductive system.

Ovulation disorders, manifesting as menstrual irregularities, are the cause of infertility in around 25% of couples who have difficulty conceiving. The World Health Organization (WHO) categorizes ovulation disorders into the following three groups:

1. Group I, i.e., disorders caused by hypothalamic–pituitary insufficiency;
2. Group II, i.e., disorders caused by eugonadotrophic eugonadic anovulation;
3. Group III, i.e., disorders caused by primary ovarian insufficiency (failure) [3,4].

The diagnostics of ovulatory infertility should cover the following aspects: patient interview with a special focus on the regularity of menstrual bleeding and ovulation, and gynecological examination, including a physical exam; in addition, ultrasound assessment of the menstrual cycle is used. The following hormonal tests are also recommended: follicle-stimulating hormone (FSH), luteinizing hormone (LH), estradiol, thyroid-stimulating hormone (TSH), testosterone, progesterone, and prolactin. In clinically justified situations, in order to rule out diminished ovarian reserve, anti-Müllerian hormone (AMH) concentration tests should be performed [5].

Other very important aspects must be considered in the context of infertility and these include lifestyle and environmental factors such as diet, physical activity, stress, and environmental pollution, among many others. They may have a negative or positive impact on female fertility, including on the course of ovulation [6]. Thus, research into the relationship between lifestyle/environmental factors and ovulatory infertility should focus on establishing which factors can be linked to particular ovulation disorders recognized as causes of infertility and the precise nature of those links.

The analysis of possible causes of ovulatory infertility presented in this article is based on a literature review performed by the authors and adopts the aforementioned WHO classification of ovulation disorders as the framework for conclusions. The authors made the logical assumption that the WHO classification must be exhaustive and, thus, the disorders included in it constitute all the currently known pathologies that may possibly constitute causes of ovulatory infertility. Thus, the authors stipulated that a causal link between a particular factor (i.e., a pathology or a lifestyle factors) and ovulatory infertility must be established through its relationship with one or several of the ovulation disorders included in the WHO classification. In other words, the following rationale was followed: ovulation disorders are primary causes of ovulatory infertility, while the analyzed factors play various secondary roles ranging from causative to coincidental. This rationale is reflected in the methodology of the study.

The aims of this review were (1) to perform and present a review of the literature data in the area of possible, probable, and suspected causes of ovulatory infertility; (2) to identify the role of lifestyle factors in the etiology of ovulatory infertility; (3) to propose a classification of relationships between ovulation disorders (as categorized by WHO) and lifestyle factors as causes of ovulatory infertility; and (4) to identify areas for further research.

## 2. Materials and Methods

This study was carried out as a review of the literature published from 1988 to 2023 in the following databases: PubMed/Medline, EMBASE (Elsevier), Scopus, Web of Science, and Google Scholar. The review focused on the current scientific consensus concerning the relationships between ovulation disorders, lifestyle factors, and ovulatory infertility. It should be noted that the focus of the article is on the co-dependence between ovulation disorders and lifestyle factors in the context of ovulatory infertility; thus, mainly those articles that dealt with both were included in the review. The authors made sure to include the most recent available articles; however, due to the relatively small number of articles dealing with lifestyle factors in relation to ovulatory infertility, older research was also included if it was deemed up-to-date and relevant, especially if the article in question was a systematic review or a meta-analysis. This issue was especially important in the case of Group I and Group III disorders. In the case of the former, any links, i.e., causal or associative, with ovulatory infertility are uncertain, while the nature of the etiologies of the disorders makes any contribution of lifestyle factors to their course and/or management of relatively little importance. As far as the latter are concerned, although Group III disorders are known to be connected with ovulatory infertility, any possible impact of lifestyle on this relationship is negligible. Thus, the literature data on this topic are extremely limited. It needs to be emphasized that the aforementioned scarcity of articles itself indicates a need for new original research in the area, and one of the purposes of the article is to point out the research directions.

The study also involved a comparison between the conclusions drawn from the literature review and the WHO classification of ovulation disorders with the view of compiling a novel classification of relationships between ovulation disorders and lifestyle factors as probable causes of ovulatory infertility. The WHO classification itself was chosen based on several factors. First of all, it provided a clear framework of ovulation disorders, which the authors considered as the key elements in the complex inter-relationships between the studied lifestyle factors involved in ovulatory infertility. In addition, the classification is clinically relevant and, thus, made it possible to analyze the degree of relationships between lifestyle factors and ovulatory infertility beyond the broad and often vague conclusions encountered in the literature. Moreover, based on data thus collected and their analysis, recommendations and areas for further research were proposed.

It should be noted that the original WHO classification dates back to 1973 and has been cited, used, and modified by authors ever since [7]. As WHO has not published a revision, for the purpose of this article the authors used the version that incorporates the most recent updates and has been cited in numerous other scientific articles.

As mentioned, the authors ascertained that the version of the WHO classification of ovulation disorders used for the article is the most recent one. A new classification was proposed by FIGO in 2022 [8]; however, the authors decided against adopting this one for two main reasons. First of all, it classifies the disorders based on anatomy, as opposed to endocrine factors, as in the case with the WHO classification. For this reason, the FIGO classification is less fitting from the point of view of the purpose of this article as, based on the authors’ previous research, endocrine factors are co-dependent with lifestyle factors in the context of ovulation infertility [9], and, thus, need to be given due attention. Secondly, is too early to tell whether the FIGO classification is becoming widely accepted by scientists as it has met with certain criticisms, including for its complexity and the singling out of PCOS as a distinct category [7]. This latter aspect was also seen by the authors as possibly detrimental to the purpose of the review due to the fact that emphasizing such a common and relatively well-researched disorder by treating it as a separate category might result in an unbalanced view as to which areas of research are worth pursuing. In addition, categorizing PCOS in the same group as other common ovulatory disorders within an endocrine-based system enabled the authors to review the connections with lifestyle-dependent endocrine disorders in a clearer and more meaningful manner.

For the reasons stated above, the still widely accepted and more flexible WHO classification has been used by the authors. It is also worth noting that the review performed by the authors has shown that the issue of having a widely recognized and accepted recent classification of ovulation disorders is a pressing one in the scientific community, yet the fact that the WHO one (with the authors’ own revisions) has been fitting the purpose for decades points to its robustness, clarity, and adaptability, which cannot yet be stated about the FIGO classification.

The three groups, based on endocrine factors, proposed by WHO can be characterized as follows:

Group I ovulation disorders are caused by failure of the hypothalamus and the pituitary gland. Conditions such as hypothalamic amenorrhea and hypogonadotropic hypogonadism fall into this category. Women suffering from these conditions usually have amenorrhea (primary or secondary), often referred to as hypothalamic amenorrhea, which is characterized by low levels of gonadotropins and estrogen deficiency. Group II ovulatory disorders are caused by a dysfunction of the hypothalamic–pituitary ovarian axis. This group is characterized by gonadotropin disorders and normal estrogen levels. It includes women with various menstrual cycle disorders and other causes related to hormonal imbalance occurring mainly in women with PCOS, but also in thyroid disease and endometriosis. Group III ovulation disorders are tantamount to primary ovarian insufficiency. As the name suggests, this is a primary disorder whose etiological factors vary from genetic through autoimmune to environmental.

Based on the authors’ experience with the research subject, Group II seemed the most relevant as far as lifestyle factors are concerned; moreover, as mentioned above, the disorders included in this group have been linked with various endocrine factors that are known to play a role in ovulatory infertility and have a connection with lifestyle factors. Nevertheless, while recognizing the prominence of Group II disorders, the review focused equally on all three WHO Groups and the fact that one of these stands out as more relevant to lifestyle factors than the others is merely convenient rather than decisive as far as the methodology is concerned. In other words, the methodology described above applies to all three WHO Groups of ovulation disorders.

## 3. Results and Discussion

### 3.1. WHO Group I Ovulation Disorders and Their Influence on Ovulatory Infertility

WHO Group I ovulation disorders, i.e., hypogonadotropic hypogonadal anovulation, are caused by failure of the pituitary hypothalamus. Approximately 10% of women with ovulation disorders suffer from a Group I ovulation disorder [3].

As the name suggests, the cause of idiopathic hypogonadotropic hypogonadism (IHH) is not known; however, it can be congenital, such as when it is associated with the anosmia known as Kallmann syndrome [10]. Other disorders belonging to Group I include septo-opto dysplasia, panhypopituitarism, craniopharyngioma, and Langerhans histiocytosis X [11]. Treatment of WHO Group I ovulation disorders may consist of hormone supplementation, or lifestyle interventions, i.e., weight normalization and exercise [3], in the case of hypogonadotropic hypogonadism resulting from weight loss caused by excessive physical activity.

Few articles have linked WHO Group I ovulation disorders to ovulatory infertility and only one Group I disorder is connected with a lifestyle factor, i.e., hypogonadotropic hypogonadism, as hypogonadotropic amenorrhea often develops as a result of low body weight or excessive exercise. Hence, functional hypogonadotropic hypogonadism (FHH) is associated with excessive physical activity and drastically limited calorie intake, resulting in a reduced availability of energy [12], in which case there is an inverse relationship with ovulatory infertility as a reduction in exercise and weight normalization are necessary to manage FHH.

Functional hypogonadotropic hypogonadism (FHH) can be diagnosed after excluding organic causes of hypogonadism. Generally, a low body mass index (BMI), irregular or amenorrheal periods, and/or high levels of exercise have been associated with anovulation due to hypogonadotropic hypogonadism. From the point of view of the impact of lifestyle on ovulatory infertility, women with FHH can increase their chances of regular ovulation, conception, and an uncomplicated pregnancy, by gaining weight if their BMI is lower than 19 and/or moderating the level of exercise if they engage in vigorous exercise regimes [3].

Unfortunately, the literature lacks research papers directly linking the other WHO Group I disorders, i.e., septo-opto dysplasia, panhypopituitarism, craniopharyngioma, and Langerhans histiocytosis X, to ovulatory infertility. The fact that the etiologies of the aforementioned disorders are for the most part genetic, neoplastic, inflammatory, or connected with factors such as traumatic injury or primiparity, which, although broadly lifestyle-based, can be hardly considered modifiable, it is not surprising that studies connecting Group I disorders with lifestyle factors are exceedingly rare.

An interesting aspect of WHO Group I disorders is the fact that the literature lacks a consensus on whether the disorders are indeed positively correlated with the risk of ovulatory infertility. For instance, a recent paper by Naseem et al. reports that a lack of ovulation and markers of diminished ovarian reserve before treatment may not reflect poor reproductive potential [13]. Hence, although it can be surmised—by analogy with the other WHO Groups—that ovulatory infertility may indeed occur in cases of ovulation disorders included in Group I, new research is needed in this area to prove whether this is indeed the case or whether the relationship is of a different nature, i.e., coincidental.

### 3.2. WHO Group II Ovulation Disorders and Their Influence on Ovulatory Infertility

Group II ovulation disorders are defined as dysfunctions of the hypothalamic–pituitary–ovarian axis. They affect approx. 85% of all women with ovulation disorders. Common conditions such as polycystic ovary syndrome (PCOS), hyperprolactinemia, thyroid dysfunction, and endometriosis fall into this category. Treatment options for disorders from this group consist of weight reduction, and various types of medical treatment, including laparoscopic ovarian diathermy (LOD), ovulation induction with injectable gonadotropins, and assisted conception [3].

#### 3.2.1. Polycystic Ovarian Syndrome (PCOS)

PCOS is the most common endocrine disorder and the leading cause of ovulatory infertility in women. It occurs in 5–10% of the population of women of reproductive age [14]. The Rotterdam Criteria is the most widely used classification of PCOS, now endorsed by most scientific societies and health authorities [15,16,17,18]. The Rotterdam definition suggests that polycystic ovary syndrome can be diagnosed in any woman with at least two of the following three features: clinical and/or biochemical hyperandrogenism (HA), ovulation abnormalities, and an ultrasound image indicating polycystic ovaries [19].

Polycystic ovary syndrome is a highly inherited, complex polygenic multifactorial disorder. Pathophysiological abnormalities in gonadotropin secretion or action, ovarian folliculogenesis, steroidogenesis, insulin secretion or action, and adipose tissue function, among others, have been described in PCOS. Furthermore, women with PCOS are at increased risk of developing the following disorders: glucose intolerance and type 2 diabetes mellitus; hepatic steatosis and metabolic syndrome; hypertension, dyslipidemia, vascular thrombosis, cerebrovascular accidents, and possibly cardiovascular events; obstetric complications; endometrial atypia or carcinoma and possibly ovarian malignancy; and mood and psychosexual disorders [20,21,22].

Of all women with PCOS, about two-thirds ovulate irregularly, with the chance of ovulatory infertility increasing as a result [23,24]. This indicates that PCOS is a leading factor in ovulatory infertility.

#### 3.2.2. Hyperprolactinemia

Hyperprolactinemia is the most common dysfunction of the hypothalamic–pituitary axis and is more common in women. The incidence of hyperprolactinemia ranges from 0.4% in the general adult population to as much as 9–17% in women with reproductive disorders [25]. Hyperprolactinemia causes hypogonadism, menstrual irregularities, or amenorrhea in women, low serum testosterone levels in men, and infertility and sexual dysfunction in both sexes [26]. In women, pathological hyperprolactinemia usually manifests itself as an ovulation disorder and is often associated with secondary amenorrhea and ovulatory infertility. However, cases of very high prolactin levels are not uncommon, sometimes occurring in women who experience normal ovulation and do not require any treatment. The disorder can also be asymptomatic [27,28]. Hyperprolactinemia is often accompanied by insulin resistance (IR), an important clinical problem in its own right nowadays [25]. As hyperprolactinemia is often associated with ovulatory infertility, conducting further research into whether it might constitute a direct cause of ovulatory infertility would be worthwhile.

#### 3.2.3. Thyroid Dysfunction

Thyroid disorders are the second most common endocrine conditions in women of reproductive age. By influencing the actions of folliculotropic and luteinizing hormones, thyroid hormones are involved in the regulation of the menstrual cycle and the achievement of fertility. For this reason, they affect all aspects of reproduction [29]. Moreover, severe thyroid dysfunction can lead to menstrual and ovulation disorders and ovulatory infertility through direct and indirect interactions with the hypothalamic–pituitary–ovarian axis and the reproductive organs. However, the exact incidence of infertility in women with thyroid disorders remains unknown [30]. Hence, conducting studies aimed at establishing or disproving the existence of a direct relationship between thyroid disorders and ovulatory infertility seems reasonable.

#### 3.2.4. Endometriosis

Endometriosis is an inflammatory estrogen-dependent condition associated with pelvic pain and infertility [31]. It appears to be one of the most common benign gynecological proliferations in premenopausal women since it is estimated that 10–15% women of reproductive age suffer from pelvic endometriosis [32]. The disorder affects gametes and embryos, fallopian tubes and embryo transport, and the eutopic endometrium, with all of these abnormalities likely affecting fertility. Therefore, an association between endometriosis and infertility is well supported with evidence throughout the literature, yet a definite causal relationship is still controversial [33]. Current treatment options for endometriosis-associated infertility focus either on stimulating follicle development and ovulation or inhibiting the growth and development of endometrial lesions [34].

As mentioned above, it has not been unequivocally proven that endometriosis is a disease that causes ovulatory infertility, as there are other co-occurring factors that can affect infertility in endometriosis. A relationship certainly exists, but current studies have not ascertained causality between the two conditions.

### 3.3. WHO Group III Ovulation Disorders and Their Influence on Ovulatory Infertility

Group III ovulation disorders are tantamount to primary ovarian insufficiency. Around 5% of women with ovulation disorders are affected by a Group III ovulation disorder [3] and the possibility of their management through lifestyle changes is negligible. For this reason, the literature data in this area are also scarce, i.e., although primary ovarian insufficiency is often studied in terms of its role in ovulatory infertility, the aspect of lifestyle as a modifiable factor in the management or, e.g., prevention of the disorder is mentioned only rarely.

Primary ovarian insufficiency is a subclass of ovarian dysfunctions in which the cause is within the ovary. In most cases, an unknown mechanism leads to premature exhaustion of the resting pool of primordial follicles. Primary ovarian insufficiency might also result from genetic defects such as Fragile X Syndrome (FXS) and Turner’s syndrome, as well as chemotherapy, radiotherapy, drugs, surgery, environmental factors, or autoimmune diseases. The main symptom is the absence of regular menstrual cycles, while the diagnosis is confirmed when elevated follicle-stimulating hormone levels and reduced estradiol concentrations are detected in the serum. The disorder usually leads to sterility and has a pronounced effect on reproductive health when it manifests at an early age [3].

Although, as mentioned above, the cause of primary ovarian insufficiency may lie in environmental factors, such as smoking or exposure to Endocrine Disrupting Chemicals (EDCs), Polycyclic Aromatic Hydrocarbons (PAHs), or Organophosphorus Pesticides [35], once the disorder has occurred, its management through modifications of the causative factor(s) is impossible, e.g., quitting smoking is not expected to have an impact on the condition. Thus, primary ovarian insufficiency is often thought as unrelated to lifestyle or adherence to a proper diet [36]. Studies exist, however, that suggest otherwise, i.e., that the condition could be delayed or better managed through personalized dietary and lifestyle recommendations based on genetics, with the rationale for such conclusions rooted in the possible influence of oxidative stress and inflammation postulated by the researchers [37]. Such conclusions, nevertheless, seem far-fetched and would be difficult to prove experimentally, with the study admitting that genetic causes indeed play the most important role in the development of primary ovarian insufficiency.

It should be noted that among women with ovulation disorders categorized in Group III, extremely low ovarian reserve parameters are not always associated with the absence of follicles in the ovary [38]. Spontaneous pregnancy is rare in women with WHO Group III ovulation disorders, with reports of spontaneous conception ranging from 2.2% to 14.2% [39]. The percentage of pregnancies achieved in women with Group III ovulation disorders is, thus, very low and the mechanisms by which ovarian activation strategies induce spontaneous conception are unknown. Therefore, it can be assumed that the vast majority of women with Group III ovulation disorders experience ovulatory infertility with negligible to non-existent possibilities of management of the condition through lifestyle modifications.

### 3.4. Relationship between Lifestyle Factors and Ovulatory Infertility

#### 3.4.1. Diet vs. Ovulatory Infertility

Nutrients play a number of functions in the human body, i.e., they provide energy, serve as building materials, and control body processes. They are inseparable from diet as food is the most significant and essential source of nutrients. For this reason, diet—understood as consumption of particular food ingredients and as an individual model of nutrition in the holistic sense—may have an impact on the ovulatory cycle and female infertility [40].

A study performed by Grieger et al. connected the Western diet with infertility and a slightly increased time-to-pregnancy [41]. Research performed by Chavarro et al., which focused on the influence of macronutrients on fertility, showed that the overall intake of carbohydrates and the glycemic load of a diet were positively correlated with the risk of ovulatory infertility. Among those same women, increased protein consumption was connected with an increased risk of ovulatory infertility. In addition, the increased risk of ovulatory dysfunction was mainly caused by the consumption of proteins of animal origin, which was in turn was directly connected with ovulatory infertility. Women who were characterized by the increased consumption of proteins of animal origin also consumed more saturated fatty acids compared to those who consumed less proteins of animal origin. For this reason, a potential influence of both high-carbohydrate diets with a high glycemic load and the increased consumption of saturated fatty acids need to be considered, as these factors may intensify the relationship between animal-fat consumption and ovulatory dysfunction. On the other hand, no relationship between the consumption of various fibers and the risk of ovulatory infertility has been discovered. As far as vegetable proteins are concerned, a beneficial influence on ovulation has been shown, i.e., when 5% of energy was sourced from vegetable instead of animal protein, the risk of anovulatory infertility more than halved [42].

A literature review showed that a high consumption of saturated fatty acids (SFA) and trans fatty acids (TFA) is connected with an increased risk of ovulatory infertility. The review suggested that the high consumption of TFAs (over 1% of total energy) is a risk factor of both female and male infertility [43]. There are other studies, however, that did not find a correlation between SFA consumption and an increased risk of ovulatory dysfunction [44]. On the other hand, Chavarro et al. showed that any 2% increase in the consumption of energy from unsaturated trans fatty acids compared to carbohydrates was connected with a 73% greater risk of ovulatory infertility and that sourcing 2% of energy from trans fats instead of polyunsaturated n-6 fats was connected with a similarly increased risk of ovulatory infertility [45].

Moreover, the appropriate quality and quantity of consumed fatty acids seems to be of crucial importance in terms of ovulatory infertility as insufficient contents of fats in diet may contribute to abnormal menstrual cycles [45]. In addition, results obtained by Mumford et al. showed that a high-fat diet induced increased testosterone synthesis in women, which has a negative impact on ovulation [44]. However, as far as ovulatory dysfunctions are concerned, the quality of fats in diet seems more important than their quantity. Polyunsaturated fatty acid (PUFA) ratios, particularly the omega-6 (n-6) to omega-3 (n-3) ratio may be among the most important factors.

It is becoming increasingly common in the literature to compare the “Mediterranean-type dietary pattern” with the “Western-type dietary pattern” (WDP). In a study performed by Toledo et al., a MedDP was characterized by a high consumption of vegetables, fruit, olive oil, wholegrain products, low-fat dairy and poultry, and oily fish and nuts, with a low consumption of red meat and simple sugars. WDP, on the other hand, was characterized by a high consumption of processed meats, red meat, fast-food products, eggs, full-fat dairy, highly processed products, sauces, and refined grain products, with a low consumption of fish, nuts, or olive oil. The results of the study showed a positive relationship between the adherence to a MeDP and a higher probability of establishing a pregnancy [46]. Similarly, Karayiannis et al. showed that better adherence to the Mediterranean-type diet in a six-month period before in vitro fertilization (IVF) was connected with a higher chance of achieving a clinical pregnancy and a live birth among women younger than 35 years of age [47].

Moreover, the current data concerning diet and female infertility suggest that certain dietary modifications may be beneficial in the prevention of low-grade chronic inflammation present in PCOS and might lead to improvements in reproductive outcomes in these patients by regulating the menstruation cycle and lowering the probability of ovulatory infertility. Nevertheless, it is not currently clear whether particular dietary and lifestyle modifications have a beneficial impact on patients’ reproductive outcomes. Further studies combined with the efficient collection of nutritional data from patients seeking infertility treatment would provide crucial insight into the potential role of dietary modifications in improving reproductive outcomes in women with PCOS.

Dietary modifications may have a direct influence on body mass reduction and are one of the treatment options of Group II disorders, which account for 85% of causes of ovulatory dysfunction. Due to the considerable importance of diet in the treatment of ovulatory dysfunction, it is necessary to fully determine what nutritional modifications are the most advisable in the treatment of ovulation disorders.

#### 3.4.2. Insulin Resistance (IR) vs. Ovulatory Infertility

Carbohydrate metabolism disorders are a serious and overly common problem nowadays, most often affecting people with excessive body weight or hormonal imbalances. Insulin resistance is one condition of this type that affects both female and male fertility [9]. The literature discusses associations between IR and PCOS [48,49,50,51,52,53,54,55], hyperprolactinemia [25,56], endometriosis [57], and thyroid disorders [58,59]. However, the best-studied disorder affecting ovulatory function in the context of IR is PCOS, which is, in turn, a known factor contributing to ovulatory infertility.

IR is a metabolic disorder defined as the inability of a known quantity of insulin (exogenous or endogenous) to increase glucose uptake and use of the glucose in the patient’s body to the degree that is typical for the healthy population [60]. Certain lifestyle factors such as a sedentary lifestyle, or improper dietary patterns leading to overweight or obesity have a negative impact of the sensitivity of human cells to insulin [61]. Insulin resistance is also an independent predictor of impaired glucose tolerance, type 2 diabetes [62], and cardiovascular diseases in the general population [63]. Furthermore, insulin resistance is strongly connected with obesity [64]. It is known that lifestyle modifications through combined diet and exercise have a positive impact on tissue sensitivity to insulin and glucose homeostasis in overweight persons [65,66,67,68]. In addition, such modifications also result in reduced levels of pro-inflammatory markers in the body, whose elevated concentrations are also found in the course of insulin resistance [69] as well as PCOS [70]. Thus, considering the fact that PCOS constitutes one of the causes of ovulation disorders, it seems that insulin resistance and obesity need to be analyzed as possible indirect factors contributing to ovulatory infertility associated with lifestyle choices.

#### 3.4.3. IR—PCOS vs. Ovulatory Infertility

A meta-analysis of data from 619 women in 14 studies, performed by Xing et al., showed that using insulin-sensitizing drugs had a positive influence on the frequency of menstruation, the profile of sex hormones, and metabolic parameters in overweight women and those with PCOS [54]. In addition, the results of a study performed by Lee et al. showed that Homa-IR (an indicator used to assess insulin resistance calculated on the basis of fasting insulin and glucose concentrations in serum) [71] correlated negatively with SHBG concentration (R = −0.304, *p* < 0.0001). However, no correlation with the ovary volume or the number of follicles was found [55]. The results of these studies suggest that reduced insulin resistance may have a beneficial impact on ovulatory function, thus reducing the risk of ovulatory infertility.

A study performed by Gower et al. that included 30 women with PCOS showed that a diet with reduced carbohydrate contents was connected with a 27% reduction in fasting insulin (*p* < 0.001) and a 23% reduction in serum testosterone level (*p* < 0.05) [72]. This indicates that adhering to a diet with a reduced carbohydrate content was connected with reduced insulin resistance and an improved hormonal profile in women with PCOS. This may, in turn, lead to improved ovulatory function and reduced ovulatory infertility. However, the quoted study was performed on a small sample of women; therefore, it can be expected that the conclusions formulated by the authors are preliminary, which is why performing more research in this area would be recommended.

#### 3.4.4. IR—Overweight and Obesity vs. Ovulatory Infertility

Overweight and obesity also have a direct strong connection with PCOS [73,74]. Approx. 50% of women with PCOS are overweight or obese, with most of them characterized by the abdominal phenotype, which means that the excess adipose tissue collects mainly around the abdomen [75]. Interestingly, even though obesity is not among the diagnostic criteria of PCOS, both overweight and non-overweight patients with PCOS have more visceral adipose tissue (VAT) than women without PCOS, while VAT is correlated positively with the total androgen level, suggesting that overweight plays an important role in PCOS [76]. Moreover, excess central adipose tissue is closely connected with low-grade chronic inflammation and IR, which may contribute to the increased risk of PCOS [77,78]. Furthermore, due to their relationship with PCOS, overweight and obesity seem to play a role in the etiology of ovulatory infertility, but in order to establish its precise nature, i.e., correlative, causative, or coincidental, more clinical research is needed.

A study performed by Dietz de Loos et al. in 183 women showed that changes in the proportion of body mass had a statistically significant impact on the probability of the occurrence of an ovulatory dysfunction (estimation 0.157 SE 0.030, *p* < 0.001) or hyperandrogenism (estimation 0.097 SE 0.027, *p* < 0.001), with the frequency of occurrence of an ovulatory dysfunction decreasing as a result of decreasing body mass and increasing as a result of increasing body mass. This means that a reduction in body mass alone resulted in improvements in both the diagnostic features and the PCOS phenotype [79]. These data allow us to conclude that body mass normalization alone may lead to improvements in ovulatory function and have a positive impact on ovulatory infertility.

Similarly, a study was performed by Dokras et al. in which the impact of body mass reduction on the health of women with PCOS was assessed. Three groups were compared, in which hormonal contraception or intensive lifestyle changes, or both these interventions combined, were used in order to achieve a 10% reduction in body mass. All three groups showed a marked improvement in their general health condition. The group that used hormonal contraception with the simultaneous implementation of lifestyle changes achieved the most pronounced improvement in the area of body hair, total serum testosterone level, and general physical well-being, compared to any single intervention [80]. While this finding cannot be interpreted as a direct associative relationship with ovulatory infertility, the fact that reduced body mass has a positive impact on the health of women with PCOS suggests that this same phenomenon may translate into a positive impact on ovulatory infertility; however, more research is needed in this area.

Research shows that hormonal imbalance is closely connected not only with insulin resistance but also with obesity in patients with PCOS, which suggests that these co-dependent factors may have an impact on the more complex issue of ovulatory infertility.

#### 3.4.5. IR—Hyperprolactinemia vs. Ovulatory Infertility

Numerous scientific studies have shown a link between IR and hyperprolactinemia [25,81,82,83,84,85]. Elevated levels of prolactin (PRL) are often associated with increased tissue resistance to insulin. Many scientific theories have been proposed to explain the likely mechanisms behind this phenomenon. The issue of the reciprocal relationship between hyperprolactinemia and IR is clinically important and certainly requires further research and observation.

In a 2009 study that included 16 hyperprolactinemic and 12 healthy subjects, HOMA-IR values were calculated for both groups. The baseline insulin level in patients with hyperprolactinemia was higher than in the control group (6.85 ± 4.68; 3.66 ± 0.88 microU/mL, respectively; *p* < 0.05). The mean HOMA-IR and HOMA-B values were higher in the patients compared to the control group (1.49 ± 1.30; 0.78 ± 0.27, respectively; *p* = 0.02 and 136.28 ± 72.53; 64.77 ± 23.31, respectively, *p* < 0.001). This suggests that patients with hyperprolactinemia were more resistant to insulin than the controls [85].

Another study performed by dos Santos Silva et al. evaluated the prevalence of obesity, overweight, and IR in patients with prolactinoma resulting in hyperprolactinemia, before and after treatment resulting in the normalization of prolactin (PRL). Twenty-two patients with prolactinoma completed six months of treatment. Their PRL levels normalized but no significant difference in BMI was observed. However, there was a significant decrease in the insulin resistance index (HOMA-IR) and glucose levels, as assessed by the homeostasis model [82].

The above studies suggest that an association exists between the occurrence of hyperprolactinemia and insulin resistance, which is in turn often associated with ovulation disorders. For this reason, it is worthwhile to conduct more research concerning the relationship between ovulatory infertility and hyperprolactinemia.

#### 3.4.6. IR—Thyroid Diseases vs. Ovulatory Infertility

A number of articles have shown that carbohydrate metabolism may be impaired in thyroid diseases with hyper- or hypothyroidism [86]. Moreover, several studies have shown that insulin resistance occurs in the course of hyperthyroidism, which has been associated with increased HOMA-IR, decreased Matsuda (i.e., insulin sensitivity index designed to indicate the values that are comparable to Rd (the rate of disappearance of plasma glucose) as measured by an insulin clamp (insulin infusion of 1 mU/kg per minute, corrected at an insulin concentration of 100 microU/mL) with a glucose marker [87]) and Belfior (i.e., the insulin resistance index (IRI), originally described by Belfior et al., based on the assessment of glucose and insulin levels during a 75 g glucose tolerance test [88]) indices, which clearly suggests the onset of insulin resistance [89,90,91]. Other studies have shown reduced tissue sensitivity to insulin in hypothyroidism [92,93]. Some other studies, however, do not support the above observations [94].

A number of studies suggest that severe thyroid dysfunction can lead to menstrual disorders and infertility through direct and indirect interactions with the hypothalamic–pituitary–ovarian axis and reproductive organs [30]. Insulin resistance may occur in both hypothyroidism and hyperthyroidism, which may be indirectly associated with ovulation disorders in women. Although articles on insulin resistance are conflicting in their conclusions, it would be worthwhile to perform more research in this area.

#### 3.4.7. Oxidative Stress vs. Ovulatory Infertility

Oxidative stress (OS) is a phenomenon that occurs when the systems of oxidation and anti-oxidation in the human body are imbalanced, which is connected with the presence and development of various diseases. Cigarette smoking, alcohol consumption, the consumption of processed foods, certain medications, and pesticides contained in foods are among those factors that lead to the excessive production of pro-oxidative substances in the body. In addition, factors that increase the risk of oxidative stress include air pollution, UV radiation, and prolonged stress, as well as excessive physical effort. Age is another important factor, as the mechanisms that protect the body from free radicals weaken with age [95,96,97]. OS is one of the numerous factors that play an important role in ovulatory infertility [98,99,100], mainly through its involvement in the etiology of PCOS.

A systematic review and meta-analysis from 2013 performed for 68 studies in which 4933 patients with PCOS and 3671 control patients participated showed that concentrations of several byproducts of oxidative stress were significantly elevated in patients with PCOS compared to the control group. Moreover, the meta-analysis showed that certain antioxidant markers were lowered in PCOS. Concentrations of glutathione, which plays the main protective role against oxidative stress, were reduced in patients with PCOS, compared to the control group [100]. However, the authors of the meta-analysis did not show whether the factor that causes increased OS might be obesity, which is common in PCOS, or whether OS might be independent from overweight/obesity. The above findings suggest that, irrespective of whether or not an association between co-morbid obesity and OS exists, OS may be regarded as a factor contributing to ovulatory infertility through its etiological role in PCOS.

A literature review performed by Wenqian et al. discusses interactions between OS and hyperandrogenism, insulin resistance, and overweight/obesity in ovulatory dysfunction in PCOS. PCOS, HA, and IR may be induced or aggravated in cases of OS imbalance. In the case of PCOS, a high-carbohydrate diet may induce OS increase, resulting in the body entering low-grade chronic inflammation, and at the same time increasing the production of androgens. It may also have an impact on the disturbed action of insulin and aggravation of IR. High insulin levels also further worsen HA. IR may also increase serum levels of free fatty acids (FFA), which, in connection with a high-carbonate diet, may increase OS. Moreover, oxidative stress interacts with HA and IR, creating a vicious circle in the emergence and progression of PCOS [99]. In addition, overweight and obesity, which often occur in PCOS, also contribute to the development of OS and low-grade chronic inflammation [101]. Hence, it can be concluded that hyperandrogenism, IR, and obesity all constitute indirect factors that contribute to ovulatory infertility through their complex associations with PCOS.

Apart from its role in PCOS-related ovulatory infertility, oxidative stress is also linked with another WHO Group II disorder, i.e., endometriosis. A systematic literature review discussing the effects of OS on endometriosis confirms that it negatively affects fertility in women with endometriosis. OS can also affect various physiological functions, such as oocyte maturation, ovarian steroidogenesis, ovulation, and embryo implantation. An imbalance between pro- and antioxidant mechanisms leads to oxidative stress in the peritoneal milieu, follicular fluid, and ovarian environment, which may partially explain endometriosis-associated infertility [102].

Furthermore, oxidative stress is increasingly often linked with thyroid disorders [103,104,105,106]. It has also been shown that thyroid dysfunction may co-exist with ovulation disorders [30]. However, many of the mechanisms involved in the development of thyroid pathology are still unknown. Yet, a noticeable association exists between increased pro-oxidant production and oxidative damage, and the development of thyroid disease. In addition, thyroid disorders might also initiate or increase the release of reactive oxygen species (ROS) and, thus, exacerbate oxidative stress, leading to increasing oxidative damage [107]. Since thyroid diseases are associated with oxidative stress, it can be inferred that in the case of concomitant thyroid diseases, fertility disorders may result from impaired ovulatory function and ovulatory infertility caused by exposure to oxidative stress. However, the literature review performed as part of this study did not produce publications directly linking oxidative stress to ovulatory infertility.

Similarly, no studies could be found linking exposure to oxidative stress with the increased risk of hyperprolactinemia and a concomitant increased risk of ovulatory infertility. A single study could be produced, however, whose findings indicated that chronic estradiol exposure induces oxidative stress in the hypothalamus, reducing hypothalamic dopamine levels and causing hyperprolactinemia [108]. This leads to the provisory conclusion that OS-related factors may contribute to the development of hyperprolactinemia, which is associated with insulin resistance, ovulatory dysfunction, and ovulatory infertility, but more research is doubtlessly needed in this area.

It should also be mentioned that a causative connection has been suggested between increased levels of oxidative stress and primary ovarian insufficiency [37], a WHO Group III disorder. However, the study is of a very speculative character and involves a large number of confounded variables, which is why to be considered clinically relevant, its conclusions would need a corroboration in the form of a sound experimental study. What is worth noting, however, is that lifestyle factors are being studied in the context of primary ovarian insufficiency, even though the results may prove useful mainly in the area of prevention as—due to the mainly genetic etiology of the disorder—once a condition has occurred, any lifestyle modifications would have a negligible impact on its course.

On the basis of the studies discussed above, it can be inferred that a mutual interaction between the ovulatory function and OS exists mainly in WHO Group II disorders and has an impact on ovulatory infertility. Correcting oxidative stress imbalances by reducing adipose tissue and/or introducing modifications in medications, exercise, and/or lifestyle may have a beneficial impact on these disorders. At this stage, however, due to the insufficient number of conclusive studies, controversies concerning the influence of oxidative stress on ovulatory infertility still persist.

#### 3.4.8. Sleep vs. Ovulatory Infertility

Although sleep is an important component of normal physiology, sleep disturbances are a common occurrence in today’s society. Abnormal sleep patterns are connected with health conditions and co-morbidities such as obesity, hypertension, diabetes, depression, and a low quality of life [109]. Moreover, a disrupted circadian rhythm may be linked to menstrual disorders [110], which may in turn lead to ovulatory dysfunction and ovulatory infertility.

A study performed by Eisengerb et al. showed that sleep duration of <6 h (6.1% vs. 2.7%; *p* < 0.001), habitual snoring (37.8% vs. 19.0%; *p* < 0.001), and clinical sleepiness were more common in women with PCOS (12.0% vs. 8.6%; *p* < 0.026) compared to women with unexplained infertility [109]. This may indicate a positive relationship between sleep disturbances and ovulatory infertility, as opposed to idiopathic infertility. A meta-analysis performed in 2017 identified and included eight studies with adult participants and five studies involving adolescents that linked PCOS with the risk of obstructive sleep apnea (OSA). The results showed that the incidence of OSA was higher in adults (0.32; 95% CI: 0.13–0.55) compared to adolescents (0.08; 95% CI: 0.00–0.30) and that the risk of OSA was significantly higher in adult patients with PCOS (odds ratio (OR) 9.74, 95% CI: 2.76–34.41) [111]. Another meta-analysis, performed in 2022, showed that PCOS is positively correlated with the risk of sleep disturbances. The incidence of sleep disturbances was higher (OR = 11.24, 95% CI: 2.00–63.10, Z = 2.75, *p* = 0.006) in the group in which PCOS was present. Moreover, it was shown that sleepiness, as assessed on the Epworth Sleepiness Scale (ESS), was also higher in the group of women with PCOS compared to healthy women (MD = 2.49, 95% CI: 0.80–4.18, Z = 2.88, *p* = 0.004) [112].

The above results indicate a strong relationship between PCOS and OSA, as well as other sleep disturbances in adult patients. Considering the increased risk of menstrual disorders in women with disturbed circadian rhythms, it can be expected that women suffering from sleep disorders may be more prone to ovulatory dysfunction and ovulatory infertility. The literature data, however, do not allow us to connect sleep disturbances with ovulatory infertility in an unequivocal manner. Moreover, the nature of the relationship that exists between sleep disturbances and PCOS—and, thus, possibly also ovulatory infertility—is unclear, with studies leaning towards co-existence. This means that their results do not make it possible to indicate a possible causal relationship, or even a weaker type of relationship. This inference is further corroborated by other studies, which do not make a connection between sleep and PCOS. For instance, a study performed by de Sousa et al. did not show an increased incidence of obstructive sleep apnea in adolescents with PCOS, compared to healthy subjects from the control group [113].

In conclusion, although sleep disturbances seem common in PCOS, most current research is limited due to small sample sizes. What is more, no studies can be found that would link sleep disturbances with ovulatory infertility directly, which is why more research would need to be performed in the area. However, considering the greater importance of other research areas concerning ovulatory infertility, its connections with sleep disturbances should not be prioritized.

#### 3.4.9. Physical Activity vs. Ovulatory Infertility

Physical activity is an important component of lifestyle modifications. Most articles are in agreement and indicate a positive influence of physical activity on the regularity of ovulation [114,115,116,117,118].

A systematic review from 2017 showed that exercise contributed to a reduction in insulin levels and free androgens in overweight and obese women, resulting in a restoration of regulated ovulation. Moreover, intensive exercise lasting 30–60 min./d was connected with a reduced risk of anovulatory infertility. In addition, a negative impact of physical activity on ovulation was also shown, i.e., an increased risk of anovulation in persons performing extremely intensive exercises (>60 min/d) [119]. The study indicates that persons who exercise intensively and are at increased risk of anovulation are more prone to ovulatory infertility.

In a study performed by Mario et al., women with PCOS were studied in terms of their leisure time physical activity (PA), a term that which covers routine activities such as walking as a means of transport, shopping, or moderate movement, regardless of their intensity. Active women (≥7500 steps/d) with PCOS had better anthropometric and metabolic profiles compared to same-aged women with PCOS who preferred a sedentary lifestyle (<7500 steps/d). It was shown that the level of androgens was lower in the group of active women with PCOS compared to those preferring sedentary lifestyle. Moreover, increasing PA by 2000 steps/d (regardless of the type of PA) was independently linked with a reduced free androgen index (FAI) in those women [120]. On the basis of this study, it can be concluded that increased leisure time physical activity improves the hormonal profile of women with PCOS, which may translate into improved ovulatory function and reduced risk of ovulatory infertility.

Similarly, a systematic review from 2011 showed that moderately intensive physical activity improves the regularity of ovulation, and reduces IR and body mass. In addition, these improvements do not depend on the type of exercise, its frequency, or the length of a single session [114]. It can be thus stated that the quoted study is yet another one which indicates that moderately intensive physical activity may have a beneficial influence on ovulatory function and ovulatory infertility.

As far as WHO Group I disorders are concerned, excessive physical activity is an established cause of hypogonadotropic hypogonadism [12], so management of the disease must include lifestyle changes that would lead to an increased calorie intake and moderated exercise regimens in order to improve the patient’s BMI and, thus, possibly increase the chances of pregnancy. It is worth emphasizing, though, that links between Group I disorders and ovulatory infertility are uncertain, which is why lifestyle modifications would have an impact mainly on the disorder itself.

Considering the above, by treating the effects of metabolic disturbances or preventing them—through the introduction of regular physical activity—better reproductive and cardiometabolic outcomes can be achieved in the female population.

#### 3.4.10. Supplementation vs. Ovulatory Infertility

As a balanced diet is an essential component of a healthy lifestyle, a steady supply of vitamins and minerals is important at every stage of life. However, for some groups of people, proper supplementation is absolutely crucial, e.g., women of childbearing age, primarily due to the possibility of pregnancy. Vitamin and mineral deficiencies are increasingly often being observed in young women, with deficiencies in any of such components possibly having dangerous consequences for both the mother and the child [121]. For this reason, increasingly large numbers of women are choosing to reduce these deficiencies with vitamin supplementation.

As far as consumption of micronutrients is concerned, no definitive evidence has been produced as to the role most of them play in infertility. Apart from the proven negative correlation between periconceptional supplementation with folic acid and neural tube defects [122], there are only studies confirming that supplementation with 1000 mg/d of n-3 acids [123,124,125,126], 400 IU/d of vitamin E (in both studied groups vitamin E was administered together with omega-3 fatty acids) [124,126], 200 μg/d of selenium [127,128], 4000–50,000 IU of vitamin D (higher doses of vitamin D, such as 50,000 IU, were given at 2-week intervals)(in one of the studies, vitamin D was administered together with a probiotic; in another, with n-3 fatty acid; and in the other two, without additional supplementation [129,130,131,132]), or 100–200 mg/d of coenzyme Q10 [133,134,135], had a beneficial effect on the health of women with PCOS. These results do not indicate, however, the existence of an impact on ovulatory infertility.

Inositol is a carboxylic sugar belonging to the vitamin B family. The two stereoisomers of inositol that are present in the human body are mio-inositol (MI) and D-chiro-inositol (DCI). They both play an important biological role as mediators in various actions of insulin. Inositol can be found in fruit, nuts, and beans and may be used as a dietary supplement. Around 1 g/d of inositol is consumed as part of a normal diet, but the absorption of free inositol may be inhibited by glucose. Many previous meta-analyses have indicated that MI has an impact on various endocrine parameters. A meta-analysis performed by Unfer et al. showed that patients with PCOS treated with MI had lowered testosterone and SHBG levels [136]. Another meta-analysis noted an influence of MI on the levels of SHBG, androstenedione, prolactin, and total testosterone in patients with PCOS [137]. In addition, a meta-analysis performed by Jethaliy et al. showed a significant influence of MI on the levels of androstenedione and prolactin only [138]. Meta-analyses performed by Zeng and Facchinetti et al., on the other hand, did not show a significant improvement in endocrine parameters such as the testosterone level [139,140]. However, considering the prevalence of studies which indicate that the hormonal profile can be improved through the use of MI, it is worth studying whether its supplementation may positively affect ovulatory function and reduce the risk of ovulatory infertility.

A meta-analysis from 2023, which identified twenty-six randomized controlled trials that included data from 1691 patients (806 inositol, 311 placebo, 509 metformin) showed that the risk of a regular menstrual cycle was 1.79 times higher in patients treated with inositol compared to placebo (CI: 1.13; 2.85). What is more, inositols showed equivalence compared to metformin. As far as BMI (MD = −0.45; CI: −0.89; −0.02), free testosterone (MD = −0.41, CI: −0.69; −0.13), total testosterone (MD = −20.39, CI: −40.12; −0.66), androstenedione (MD = −0.69, CI: −1.16; −0.22), glucose (MD = −3.14; CI: −5.75; −0.54), and the AUC (area under the curve) of insulin (MD = −2081.05, CI: −2745.32; −1416.78) are concerned, treatment with inositol resulted in a greater BMI reduction compared to placebo. Inositol also significantly increased the level of globulin, which binds sex hormones, compared to a placebo (MD = 32.06, CI: 1.27; 62.85). This meta-analysis suggests that inositol is a safe and effective method of treatment for PCOS. Moreover, inositols showed equivalency in terms of most of the results compared to the golden standard of treatment, i.e., metformin. However, due to the considerable discrepancies in the results of meta-analyses, it would be advisable to perform additional studies in the area [141].

Moreover, several meta-analyses showed improvements in glycemic parameters such as the fasting glucose concentration, fasting insulin concentration, glucose/insulin ratio, and HOMA after treatment with MI in patients with PCOS [136,137,139,140,142,143,144]. However, two other meta-analyses did not indicate an improvement in any of the aforementioned glycemic parameters in women with PCOS [5,138]. What is more, although inositol has been proposed as a possible PCOS treatment, studies concerning the substance have been, in fact, inconclusive. On the one hand, several meta-analyses have shown a significant improvement in either BMI or WHR in patients with PCOS after treatment with MI [138,139,140]; on the other hand, the meta-analysis performed by Unfer et al. showed a reduction in BMI, but not WHR, in patients with PCOS after treatment with MI [142]. In conclusion, despite certain promising indications that there may be a negative relationship between inositol supplementation and ovulatory infertility, the literature data are too conflicting at this point to conclude that its role is appreciatively more beneficial than a placebo.

### 3.5. Limitations of the Study

This study had several limitations, most of which were the result of its subject matter. First of all, the topics of lifestyle and diet are seldom studied in a rigorous clinical setting, with observational studies being more common. This results in a considerable uncertainty of results and a preliminary character of many of the studies. Moreover, infertility is a complex issue in itself, in terms of biology and biochemistry, as well as methodology. For this reason, most studies did not offer conclusions that would make it possible to establish clear associative links between the aspects discussed in this article. Another issue is the complex inter-relationship between lifestyle factors, diet, and ovulatory infertility, especially in terms of insulin resistance, obesity, and PCOS, which makes separating the individual factors and establishing relationships between them exceptionally difficult. In addition, the subject literature concerning ovulation disorders is very much biased towards PCOS, which is why data concerning other possible medical causes of ovulatory infertility are scarce. Furthermore, a larger number of available recent articles, especially those concerning lifestyle factors in the context of ovulation infertility, would prove beneficial for the study. Last but not least, as the study attempts to point directions in an area of research that is characterized by a certain amount of vagueness, in particular as far as diet and lifestyle are concerned, it is burdened with those same issues that other pioneering studies deal with, i.e., a large degree of uncertainty as to the stated conclusions and the need for further research—in particular robust studies performed in clinical settings—in many of the analyzed areas.

## 4. Conclusions

Since various lifestyle factors seem to have a significant impact on ovulation and the occurrence of ovulatory infertility [22,145], it is important to find relationships between the factors and establish the degrees of their impact, which could lead to a reduction in the rate of ovulation disorders. Therefore, the classification and the areas for further research presented below are intended as starting points for studies—especially robust studies performed in clinical settings—that could establish links between ovulatory infertility and the factors that coincide or form a relationship with it. Identifying such links would prove clinically significant and possibly help to improve the comfort of women who want to become pregnant, and increase the rate of spontaneous pregnancies.

### 4.1. Classification of Relationships between Ovulation Disorders and Lifestyle Factors as Probable Causes of Ovulatory Infertility

Based on the performed literature review and taking into account the existing knowledge gaps, uncertainties, and possible research directions, the authors propose a classification of links between lifestyle factors and ovulatory infertility (Table 1). The table below couples each of the lifestyle factors discussed in the previous section with a WHO disorder (or several disorders), in accordance with the analyzed literature data. The WHO Group that the particular disorders belong to are given in brackets. Moreover, the degree of the relationship between a particular factor and ovulatory infertility is established in each case. The classification naturally largely focuses on WHO Group II disorders, which is the result of both their highest incidence and their prominence in published research. As the influence of lifestyle factors on ovulatory infertility in WHO Groups I and III seems negligible, the table reflects this state.

### 4.2. Areas for Further Research

Firstly, it should be noted that the relatively limited number of recent studies concerning the precise role of lifestyle factors in ovulatory infertility points to the existence of a broad research area that could benefit from well-designed clinically relevant studies, ideally focused on the directions indicated below.

When analyzing the results of the literature review and the proposed classification presented above, the authors paid special attention to applying a highly critical approach to identifying possible areas for further research. First of all, the aspect of clinical significance was regarded as a priority. In other words, those interventions that may feasibly lead to improvements in clinical practice and patient outcomes concerning ovulatory infertility were identified as benefitting further studies. Thus, in order to avoid the common mistake of indicating any area where data are inconclusive as worthy of further research, the authors used their varied expertise in fertility medicine, nutrition, and critical thinking as applied to research. Hence, care was taken to single out those areas in which performing further studies may in fact constitute a waste of resources due to the extremely low probability that the results of such studies would have a noticeable impact on the clinical practice in such areas. Consequently, the authors feel that greater attention should be paid to those issues that indeed show promise as far as clinical applications are concerned.

Both hyperprolactinemia [26,27] and thyroid disorders [29] can undeniably result in irregular menstruation and ovulation disorders. They have also been connected to some degree with ovulatory infertility [27,28,31]. However, they have not been proven to constitute direct causes of ovulatory infertility due to the considerable proportion of spontaneous pregnancies that occur in the course of these disorders [27,28,30]. Moreover, both hyperprolactinemia [25] and thyroid disorders [146] are accompanied by insulin resistance to a greater degree in women experiencing infertility issues than in healthy individuals. It is also known that insulin resistance can be a major problem in the case of ovulatory infertility [147,148,149,150]. Given the close relationship between symptoms associated with both hyperprolactinemia and thyroid disorders and ovulatory infertility, it would be advisable to study the relationships in terms of possible causal relationships.

As far as endometriosis is concerned, the condition affects the fallopian tubes, causing abnormalities in their function that can definitely affect ovulatory infertility. Although associations between endometriosis and infertility are well supported in the literature, any specific causal relationship is still controversial [33]. In addition, studies are unlikely to link endometriosis with insulin resistance, despite the fact that the condition often co-occurs in the course of ovulation disorders. For this reason, this direction of research does not seem especially promising. Nevertheless, given the high prevalence of endometriosis in women of childbearing age and the morbidity associated with it, it would be worthwhile to conduct more studies concerning the direct cause of ovulatory infertility in the context of endometriosis.

In terms of insulin resistance, it cannot be conclusively stated that it constitutes a direct cause of ovulatory infertility. Above all, insulin resistance itself is a secondary condition, with causes ranging from improper diet to insufficient physical activity. What makes insulin resistance a matter worth investigating in the context of ovulation fertility, though, is its relationship not only with the aforementioned lifestyle factors but also with PCOS [9,150]. The relationship appears to be a particularly complex one and the authors feel that studies concerning its exact nature may produce valuable insights into the issues of both the causes and effects of ovulatory infertility—as well as insulin resistance and PCOS. At this stage, what appears to be fairly certain as far as research directions are concerned is that the aspect of causality is particularly worth exploring.

An inadequate diet significantly increases the risk of obesity, which is associated with a number of adverse effects on the mother and the fetus during the prenatal period. Moreover, it has a negative impact on female fertility, i.e., obese women are more prone to ovulation disorders due to dysregulation of the hypothalamic–pituitary–ovarian axis [151]. It has also been shown that following the obesity-promoting Western diet, rich in saturated fats, animal protein, and simple sugars, is associated with an increased risk of ovulatory infertility [41,42,43,45]. Furthermore, it has been well established that obesity plays a key role in the onset of insulin resistance [150,152], which often co-occurs with ovulation disorders. Thus, weight reduction is one option of treatment for ovulation disorders. There are no studies, however, that would establish a direct link between an inadequate diet and the development of ovulatory infertility. Given the high incidence of obesity-related problems, it would be worthwhile to conduct more studies examining the direct link between obesity/improper diet and ovulatory infertility.

Interestingly, physical activity may have a bidirectional effect on ovulation disorders. Some studies have shown that intense exercise of 30–60 min./d is associated with a reduced risk of anovulatory infertility. In contrast, an increased risk of anovulation has been reported in women exercising very intensively (>60 min/d), who are often co-morbidly underweight, with impaired HPA function [119]. Therefore, it can be assumed that excessive physical activity may lead to ovulation disorders and, consequently, ovulatory infertility. However, more research on the role of long-term/intensive training and chronic energy deficit in the context of ovulatory infertility is needed.

Physical activity is also a factor present in research concerning WHO Group I ovulation disorders. It has been established that lifestyle modifications leading to weight reduction, especially exercise, have a beneficial impact on hypogonadotropic hypogonadism [3,12]. Hence, further research could be performed to replicate the earlier results and link them directly with an impact on ovulatory infertility, which is still very uncertain. Moreover, studies could be attempted at establishing links with other lifestyle factors and other Group I disorders, especially when other modifiable factors are concerned.

Although sleep quality appears to be a factor associated with ovulatory infertility, the literature data do not provide a definite link between sleep disorders and ovulatory infertility. Moreover, the degree of relationship between sleep disorders and ovulatory infertility is unclear. Its uncertain nature, discussed earlier in this article, has been further confirmed by other studies that have shown no association between sleep and PCOS [113]. Therefore, sleep quality is probably a factor that does not merit further investigation in relation to ovulatory infertility, or should at least be prioritized lower than more clinically relevant areas of research.

Similarly, despite the fact that oxidative stress seems to have a significant impact on ovulatory disorders, there is much inconclusive research published on the topic, while the phenomenon of oxidative stress itself is a very broad research area [100]. This is why it would be worthwhile to conduct studies focused on specific oxidative stress triggers, which could narrow down the topic and lead to results that would be clinically applicable. OS has also been studied in the context of WHO Group III ovulation disorders, i.e., primary ovarian insufficiency, with a causative link proposed by the researchers [37]. This tentative finding, based on a relatively new idea, could be examined further, provided that a robust clinical study could be designed. However, due to the large genetic component to the etiology of primary ovarian insufficiency and the large number of confounded variables, this task could prove extremely difficult.

Inositol supplementation might be a promising method of treatment for ovulatory infertility [141]. However, since the literature data are too conflicting at this point to conclude that its role is more beneficial than a placebo, more high-quality studies on this topic would need to be conducted to confirm these preliminarily suggested relationships.

As far as Group I and Group III disorders are concerned, due to the fact that they have seldom been researched in the context of lifestyle factors and ovulatory infertility and despite the low probability of finding strong relationships, any studies that could confirm or disprove what are, at this moment, educated assumptions or hypotheses would be worthwhile as they could possibly fill some of the existing knowledge gaps in the area.

It is worth emphasizing that based on the viability of the areas for research indicated in the previous chapter, clinical and observational studies aimed at establishing the precise nature and degree of relationship between lifestyle factors and ovulatory infertility could be designed and performed. Obviously, the nature of infertility as a research problem makes it difficult to collect robust conclusive data; however, when taking into consideration the results of published studies, certain promising directions of research can be established, as well as those that do not appear to be worth pursuing.

## Figures and Tables

**Table 1:** Classification of relationships between ovulation disorders and lifestyle factors in ovulatory infertility.

Lifestyle Factor	Possibly Related Disorder from WHO Classification (Group)	Degree of Relationship with Ovulatory Infertility
Diet	PCOS (II)	Possible positive/negative relationship Co-dependence with obesity and insulin resistance
Insulin Resistance	PCOS, hyperprolactinemia, thyroid dysfunction (II)	Possible positive relationship Co-dependence with diet and obesity
Oxidative stress	PCOS, hyperprolactinemia, thyroid dysfunction, endometriosis (II) Primary ovarian insufficiency (III)	Probable positive relationship (degree of relationship varies depending on the disorder) Co-dependence with insulin resistance Possible weak causal relationship
Sleep	PCOS (II)	Unknown, possible association
Physical activity		Possible negative relationship
	PCOS (II)	Possible weak positive relationship
		Co-dependence with obesity
	Hypogonadotropic hypogonadism (I)	Possible negative relationship
Supplementation	PCOS (II)	Negligible

## Data Availability

Not applicable.
